# Dynamic Image Difficulty-Aware DNN Pruning

**DOI:** 10.3390/mi14050908

**Published:** 2023-04-23

**Authors:** Vasileios Pentsos, Ourania Spantidi, Iraklis Anagnostopoulos

**Affiliations:** School of Electrical, Computer and Biomedical Engineering, Southern Illinois University, Carbondale, IL 62901, USA

**Keywords:** Deep Neural Networks, pruning, embedded systems, image difficulty

## Abstract

Deep Neural Networks (DNNs) have achieved impressive performance in various image recognition tasks, but their large model sizes make them challenging to deploy on resource-constrained devices. In this paper, we propose a dynamic DNN pruning approach that takes into account the difficulty of the incoming images during inference. To evaluate the effectiveness of our method, we conducted experiments on the ImageNet dataset on several state-of-art DNNs. Our results show that the proposed approach reduces the model size and amount of DNN operations without the need to retrain or fine-tune the pruned model. Overall, our method provides a promising direction for designing efficient frameworks for lightweight DNN models that can adapt to the varying complexity of input images.

## 1. Introduction

Deep Neural Networks (DNNs) have emerged as a popular choice for solving various challenging machine learning tasks, especially in the field of computer vision. Inside a DNN, matrix multiplication is a fundamental operation that forms the backbone of many common network layers, such as fully connected and convolutional layers. These operations are typically implemented using multiply accumulate (MAC) units, which perform both multiplication and addition in a single operation [1]. While MAC operations are essential for DNNs to function accurately, they also contribute significantly to the elevated energy consumption of these networks [2,3].

Therefore, even though DNN models have demonstrated remarkable accuracy on different image recognition benchmarks, their high computational and memory requirements limit their deployment on resource-constrained devices such as embedded systems. A commonly used technique is pruning, where the weight tensors of a DNN are modified to be sparse [4]. This approach involves removing a subset of network parameters, such as weights or neurons, that are deemed unnecessary or redundant. By reducing the number of parameters, DNN pruning reduces the number of MAC operations required for inference, thereby decreasing the energy consumption of the network. However, this removal of parameters from the original DNN model can lead to drops in the final accuracy, which is usually recovered through additional retraining or fine-tuning on the pruned model [5].

Previous research on DNN pruning has proposed various techniques for lightweight models. An approach is to zero weights whose magnitude exceeds a specified threshold [6]. A more aggressive approach is the removal of entire filters and neurons [7]. Instead of pruning individual weights, the work in [8] prunes groups of weights through the introduction of depth-wise and shape-wise sparsity instead. Through reinforcement learning, the work in [9] prunes convolution channels. To recover from the pruning-induced accuracy loss, these works fine-tune and retrain the compressed generated models. However, retraining is not always a feasible task. The required dataset might not even be available, or there might be privacy issues [10]. At the same time, retraining on embedded devices is a very time-consuming and computationally-intensive procedure [11].

In this work, we present a pruning framework that takes into account the difficulty of the input image during inference. In contrast to related works, we do not employ extensive offline explorations or retraining, and at the same time, we allow for a more data-driven and dynamic selection of pruning combinations. Image difficulty refers to the level of challenge posed by an input image for a DNN. Different images may require different levels of network complexity to achieve high accuracy. Some images may be relatively easy to classify, while others may be more challenging due to factors such as occlusion, lighting, or complexity. However, the term image difficulty can be subjective and arbitrary, and previous works have relied on human feedback alone to categorize an image as difficult for classification [12]. However, human perception may not always be an accurate measure of image difficulty, as DNNs extract features that are not visible to the human eye and process image information in a different way. Therefore, it is necessary to develop objective metrics that can quantify image difficulty and accurately assess the network’s performance on different input images.

Overall, we propose a framework that:

1. Utilizes difficulty metrics that incorporate human observations as well as image quality scores to build a prediction model that can predict the difficulty of an image at run-time;
2. Introduces a lightweight exploration loop for pruning combinations; and
3. Adaptively prunes DNNs during inference time based on the predicted image difficulty scores.

## 2. Methodology

This section will present the proposed framework and its different components. First, we will present our employed metric for image difficulty and its utilization in a Support Vector Machine (SVM) classifier model. Afterwards, we will show an exploration loop that generates a pool of candidate pruned DNN models, and finally, we will present how to combine the SVM classifier and the pruned DNN models at run-time to achieve lightweight inference.

### 2.1. Quantifying and Classifying Image Difficulty

For this part of the methodology, we employed the minimum required viewing times dataset [13]. This dataset assesses the time required by human subjects to recognize objects from the ImageNet and ObjectNet datasets. The dataset provides valuable insights into how long it takes humans to classify images. We propose that this human-based information can be used toward building an effective prediction model. By analyzing the dataset in [13], we can identify patterns and characteristics of images that are easy or difficult for humans to classify. Intuitively, an image that requires a longer viewing time to classify is likely to have more complex features, occlusions, or ambiguities that make it challenging for humans to recognize. On the other hand, an image that can be classified quickly likely has clearer and more distinctive features that make it easier for humans to identify. Alongside the viewing times dataset, the work in [13] also proposed two difficulty metrics. The first metric is a difficulty score, which measures the proportion of humans who were able to correctly classify a given image. The second metric is the minimum amount of time required to reliably classify an image. This metric takes into account the time taken by the participants to accurately recognize the image, with longer times indicating greater complexity and difficulty.

To that end, we propose an image difficulty metric that combines both of these aspects into a single value. Therefore, for an image *i*, we define the difficulty as follows:

(1) $${Difficulty}_{i}=\frac{Correct\_Classifications}{Total\_Participants}\times \left(1-\frac{{Avg\_Time}_{i}-min\left({Time}_{total}\right)}{max\left({Time}_{total}\right)-min\left({Time}_{total}\right)}\right)$$

In Equation (1), the fraction $\frac{Correct\_Classifications}{Total\_Participants}$ corresponds to the number of correct classifications observed out of all of the participants. ${Time}_{i}$ is the average response time spent by all human subjects on the classification of image *i*, and ${Time}_{total}$ is a list that contains all ${Time}_{i}$ values for each image *i* recorded in the viewing times dataset [13]. With this metric, a value near 0 would mean that it is highly likely the specific image lies in the most difficult group of images in the dataset, while a value closer to 1 would indicate that the image was rarely misclassified and could be deemed an easier one. At this point, we want to mention that we do not conduct any further experiments on human subjects but solely rely on the dataset found in [13]. We use the recorded values from the participants as our only indicator of human-measured image difficulty.

**Figure 1:**
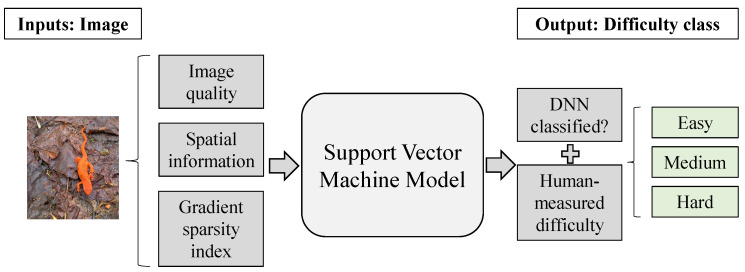
The SVM model to predict image difficulty of an input image for a target DNN.

We use the image difficulty metric defined in Equation (1) to build an SVM model that classifies any input image into one of the following three classes: Easy, Medium, and Hard. An overview of the SVM training process is shown in Figure 1. The input image is analyzed for its image quality (BRISQUE) [14], spatial information (SI) [15] and Gradient Sparsity Index (GSI) [16] metrics, which are used as the input to an SVM model with a polynomial kernel. As the output of the SVM, we combine two different pieces of information to acquire the final class: the difficulty metric defined in Equation (1) and the information of whether the DNN classifies correctly or not the image which can be obtained by running a single inference on the input image. Note that this procedure is being performed only for the training of the SVM predictor. Therefore, the final metric we use as the utilized difficulty is defined by:

(2) $${Difficulty}_{i}=0.8*\frac{Correct\_Classifications}{Total\_Participants}\times \left(1-\frac{{Avg\_Time}_{i}-min\left({Time}_{total}\right)}{max\left({Time}_{total}\right)-min\left({Time}_{total}\right)}\right)+0.2*DNN\_Classification$$

The DNN_Classification value in Equation (2) will either be 1 or 0 depending on whether the DNN can correctly classify the input image or not. We include the DNN’s ability to classify the image to have a well-rounded difficulty metric that includes not only human perception abilities but also takes into consideration how difficult the image is from the DNN’s perspective. However, we still consider the human-measured metric to be the most significant in terms of importance.

Finally, instead of employing the SVM for a regression task where it would try to map an image’s metrics to a single difficulty value as defined in Equation (2), we simply divide all the difficulty values we observe for the dataset in [13] to three equally sized categories (Easy, Medium, and Hard), and we use the SVM to classify the input image to one of these three classes. Therefore, at run-time, where we do not have the knowledge of the DNN’s classification score for a previously unseen image, the SVM will simply return a classification score which will be either Easy, Medium, or Hard.

### 2.2. DNN Pruning Tolerance

As mentioned in Section 1, related works that utilize pruning toward more efficient DNN inference usually employ extensive explorations that also require retraining. In this framework, however, one of our main goals is to prune DNNs while completely avoiding retraining and further fine-tuning.

**Figure 2:**
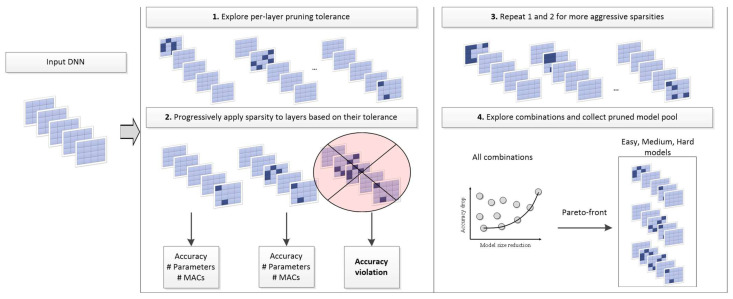
Overview of the pruning combination explorations on a given pretrained DNN model. No retraining is involved in this procedure, and the final outcome is three different combinations that will be invoked as the Easy, Medium, and Hard models.

An overview of this step is shown in Figure 2. During the pruning stage, we avoid fine-grain time-consuming explorations and initially employ layer-wise pruning, i.e., we apply pruning, and specifically channel pruning [17] at a 10% consistent sparsity to one layer at a time. The proposed strategy involves adding pruning to each layer of the DNN individually, starting with the first layer and moving toward the last (Figure 2 ①). After pruning each layer, the accuracy of the resulting network is evaluated on the validation set of the target dataset, and the respective accuracy is monitored. This process allows us to evaluate the impact of pruning each layer of the DNN in isolation. This helps us to identify the relative importance of each layer and its contribution to the overall accuracy of the DNN.

Once each layer has been pruned individually, we can determine the resilience of each layer to pruning based on its accuracy drop. The layer with the least accuracy drop is considered the most resilient, while the layer with the highest accuracy drop is considered the least resilient. We then add pruning back to the network one layer at a time, starting with the most resilient layer and moving toward the least resilient (Figure 2, ②). Every combination is being evaluated on the validation set of the target dataset, and we monitor not only the accuracy drop but the reduction in parameters and MAC operations this time as well.

So far, steps ① and ② as described above and shown in Figure 2 correspond to a 10% sparsity ratio. For step ③, we repeat steps ① and ② for 20% sparsity. Note that we have already tested the layers for resilience in step ①, so their resilience is going to be similar if not identical for more aggressive sparsity ratios, too. Then, starting from the most resilient layer to the least resilient one, we swap the initially set 10% pruning ratio to 20% until an accuracy threshold is violated. We then take the Pareto front of all generated combinations and keep only three based on the reduction in the DNN’s model size. All final three selected combinations should lie on the Pareto front and have increasing accuracy and model size reduction and will be used as the Easy, Medium, and Hard models during run-time.

### 2.3. Bringing It All Together: Run-Time Pruning Based on Image Difficulty

We combine the approaches presented in Section 2.1 and Section 2.2 to achieve lightweight DNN inference without compromises in terms of accuracy at run-time. We consider run-time scenarios where real-time performance is prioritized [18] and therefore target DNN inference where each image is processed independently rather than in big batches.

**Figure 3:**
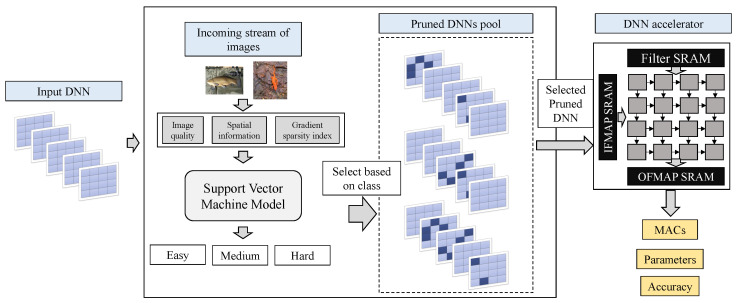
The execution flow of the proposed framework at run-time. For a target DNN model, each image during inference is being analyzed for its metrics and classified through the SVM. Then, the respective DNN model is selected for inference on a DNN accelerator.

Figure 3 shows how we combine the SVM from Section 2.1 with the pruning pool resulting from the exploration described in Section 2.2. For each incoming image in the input stream, we calculate the metrics that are needed by the SVM, which then classifies it as Easy, Medium, or Hard. From the pruned models pool that we have generated in Section 2.2, we select the respective model for inference based on the verdict of the SVM, and finally, we evaluate it on a DNN accelerator. We keep the resulting accuracy, model parameters, and total MAC operations to evaluate our model in Section 3.

## 3. Evaluation

The human-based dataset presented in [13] targets the ImageNet dataset which we also employ in this work [19]. The ImageNet dataset is a widely recognized benchmark in the field of computer vision and deep learning. It consists of more than a million images distributed over 1000 categories, making it a comprehensive and diverse dataset for evaluating the performance of DNNs. The accuracy achieved on this dataset by DNNs has become a standard metric of their evaluation. We evaluate the proposed framework on the entire validation dataset of ImageNet which is 50,000 images in total. We used pretrained models on the ImageNet from Pytorch [20], which is a popular open-source machine learning library heavily used in the development of DNNs. We evaluate a variety of DNNs with diverse structures which are the following: ResNet18 and ResNet50 [21], VGG11 and VGG16 [22], AlexNet [23] and GoogLeNet [24]. We use channel pruning [17] as the employed pruning technique and evaluate DNN inference on the systolic accelerator simulator in [25]. We simulate a systolic array following the Eyeriss [26] architecture paradigm, where the input feature map (IFMAP), filter, and output feature map (OFMAP) SRAM sizes are 108 kB, and the array has a height of 12 and a width of 14.

It is important to note that the aim of this paper is not to propose a new pruning method but utilize an already proposed approach such as channel pruning. In the case of channel pruning, there are various methods for retraining the pruned model to recover the lost accuracy, such as fine-tuning the model on the pruned architecture or using knowledge distillation to transfer the knowledge from the original model to the pruned model [27]. However, these methods can be computationally expensive and time-consuming, and they may not be feasible or practical in all scenarios [18]. On the contrary, the proposed method does not involve retraining or fine-tuning; therefore, to keep evaluations fair, we avoid directly comparing our results with other pruning methods that do involve such techniques. Instead, we provide comparisons in terms of the efficiency and effectiveness of the proposed methodology based on the compression achieved alongside the drop in final inference accuracy.

Our proposed approach is intended for lightweight Convolutional Neural Networks (CNNs) utilized in image classification tasks. While we evaluate our approach on several state-of-the-art CNNs on the ImageNet dataset, it can be applied to any CNN architecture. It is important to note that our evaluation is limited to the image classification task and convolutional neural networks. However, the same principles of layer sensitivity to pruning ratios can be applied to other neural network architectures. The ImageNet dataset is widely used in the computer vision community due to its large size and complexity, and evaluating on this dataset provides valuable insights into the effectiveness of our approach.

Table 1 contains all the results of the conducted evaluation of the proposed framework. We can observe that there are three different behavior patterns based on the type of DNN deployed with our framework.

**Figure 4:**
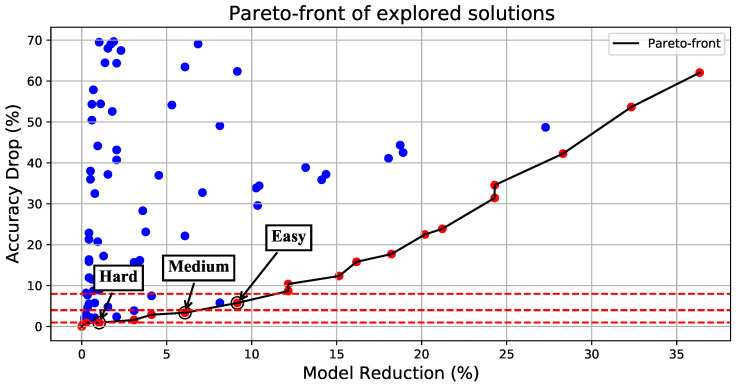
A visualization of all the explored pruning combinations of ResNet-18 alongside the underlying Pareto frontier. We select the best combination (maximum number of parameters reduced) for accuracy drops less than 1%, 4% and 8% for the Hard, Medium, and Easy DNNs, respectively.

1. The ResNet models: The ResNet models are overall more tolerant to pruning, allowing for significant speedups and a reduction in MAC operations and model size. Both of the examined ResNets comprise multiple convolutional layers, allowing for more pruning combinations to be explored. An illustrative example on the ResNet-18 specifically is shown in Figure 4. For all the combinations, we only consider solutions that lie on the Pareto front, and we select three different accuracy drop thresholds of increasing aggressiveness to select the Hard, Medium, and Easy DNNs: 1%, 4% and 8%, respectively. This way, we aim to have an overall average accuracy that does not fall more than 4% below the baseline accuracy on average. As shown in Table 1, we manage to stay below a 2% drop in accuracy for all DNNs considered in this work due to the adaptive nature of the framework at run-time.
2. AlexNet and GoogLeNet: Both of these models have very different structures. AlexNet has a small amount of convolution layers (just 8 in total), but its model requires tens of millions of parameters (61.1M) as opposed to the GoogLeNet model that has 22 convolution layers but requires just 6.62M parameters. The parameter amount for each DNN was calculated through [17]. Even though different in structure, convolution layers and parameters, they exhibit the same behavior, which is fairly conservative: they do not tolerate pruning as well, and the acceptable solutions found by the proposed framework led to lower reductions in MAC operations and parameters, which however came with a lower drop in the average accuracy.

We additionally evaluated two VGG models, as shown in Table 2. Even though the observed accuracy drops for the VGG-11 and VGG-16 models are similar to those of the ResNets, the reduction in parameter size and MAC operations is not as significant as the ResNet models. This is attributed to the fact that the VGG models comprise a smaller number of convolutional layers, which are however of higher depth. Therefore, applying a 10% sparsity to the multiple convolution layers can significantly lower the model size, which can lead to drastic drops in accuracy, as shown in the first two lines of Table 2. Therefore, for these two cases only, instead of following the procedure described in Section 2.2 with an initial sparsity of 10% followed by the more aggressive 20%, we started the procedure with a 5% sparsity ratio followed by 10%.

Overall, the proposed framework always produced final solutions that achieved speedups compared to the baseline execution due to the reduction of MAC operations and parameters without surpassing 1.85% accuracy drop from the baseline. Therefore, this evaluation indicates that incorporating image difficulty and incoming data attributes during pruning can provide several benefits. Specifically, by pruning more aggressively on easy images, we can save more computational resources without sacrificing accuracy on the most challenging samples.

### Cost Efficiency Analysis

The utilized SVM comprises a third-degree polynomial kernel and is only trained once. The main reason why the SVM is efficient for run-time deployment is that it can quickly classify new data points based on the decision boundary learned during training. To classify a new image at run-time, the SVM computes the dot product between the image’s features and the weights of the support vectors. The resulting values are then passed through a decision function that maps them to the predicted class label. This process is relatively fast since it involves only a few computations and does not require iterative training steps.

The utilized SVM is only used to classify images into three difficulty levels (Easy, Medium, and Hard) at run-time, the classification time is expected to be very fast, and it should not be a significant bottleneck in the overall inference process. Additionally, computing the necessary inputs for the SVM (image quality, spatial information, and gradient sparsity index) is in the range of microseconds.

In our evaluation, we found that the additional time required to perform SVM inference and image property calculations was negligible compared to the overall time required for DNN inference. We observed that even with the added SVM inference step, the pruned DNNs still resulted in significantly faster inference times compared to the baseline unpruned DNNs. None of the evaluated DNNs showed a final inference time similar to the baseline, indicating that the pruning of the DNN more than compensated for the minimal overhead of SVM inference.

## 4. Conclusions

Deep Neural Networks (DNNs) are becoming increasingly complex, making them difficult to deploy on resource-constrained devices. While pruning mechanisms can result in lightweight DNNs, they typically require additional retraining to recover from accuracy losses induced by pruning. This paper proposes a dynamic DNN pruning approach that takes into account the difficulty of incoming images during inference on pretrained DNNs. Experimental results on the ImageNet dataset demonstrate that the proposed approach effectively reduces model size and DNN operations without the need for retraining or fine-tuning on the pruned model, resulting in speedups during inference. By considering the attributes of the input data, the proposed approach presents a promising direction for improving the deployment of DNNs on resource-constrained devices.

## Figures and Tables

**Table 1:** Evaluation results.

	Accuracy Drop (%)	MAC Reduction (%)	Parameter Reduction (%)	Speedup
ResNet-18	1.84	12.11	9.65	×1.28
ResNet-34	1.75	11.53	9.41	×1.36
ResNet-50	1.79	11	9.32	×1.35
AlexNet	0.58	5.61	4.23	×1.1
GoogLeNet	0.78	7.88	5.82	×1.15

**Table 2:** Evaluation results for VGG networks: At first, with no alterations in the initial pruning ratio (PR) (10%) and then with the adjusted initial PR (5%).

	Accuracy Drop (%)	MAC Reduction (%)	Parameter Reduction (%)	Speedup
VGG11 (10% PR)	3.25	8.4	6.6	×1.22
VGG16 (10% PR)	4.13	9.1	8.91	×1.26
VGG11 (5% PR)	1.61	6.67	3.5	×1.08
VGG16 (5% PR)	1.42	7.32	6.68	×1.14
